# RUNX2 as a novel biomarker for early identification of patients progressing to advanced-stage mycosis fungoides

**DOI:** 10.3389/fonc.2024.1421443

**Published:** 2024-10-07

**Authors:** Maria Danielsen, Thomas Emmanuel, Morten Muhlig Nielsen, Lise Maria Lindahl, Maria Gluud, Niels Ødum, Line Raaby, Torben Steiniche, Lars Iversen, Rikke Bech, Terkild Brink Buus, Claus Johansen

**Affiliations:** ^1^ Department of Dermatology, Aarhus University Hospital, Aarhus, Denmark; ^2^ Department of Molecular Medicine (MOMA), Aarhus University Hospital, Aarhus, Denmark; ^3^ Skin Immunology Research Center, Department of Immunology & Microbiology, University of Copenhagen, Copenhagen, Denmark; ^4^ Department of Pathology, Aarhus University Hospital, Aarhus, Denmark

**Keywords:** mycosis fungoides, digital spatial profiling, biomarkers, RNA profiling, spatial transcriptomics

## Abstract

**Introduction:**

The majority of patients with mycosis fungoides (MF) have an indolent disease course, but a substantial fraction (20-30%) of patients progress to advanced stages – usually with a grave prognosis. Early differentiation between indolent and aggressive types of MF is important for the choice of treatment regimen and monitoring of the individual patient. Good biomarkers are therefore desired.

**Methods:**

Here, we used spatial transcriptomics on skin samples at time-of-diagnosis to enable prediction of patients who later progressed to advanced stages of MF. Formalin-fixed, paraffin-embedded skin biopsies at time of diagnosis from six patients with MF who progressed to advanced stages of disease within 4 months to 12 years after diagnosis, and nine patients who remained in early-stage disease over 9 to 27 years were analyzed using the GeoMx Digital Spatial Profiler to capture spatially resolved high-plex RNA gene expression data. Five different regions of interest (the epidermis, the basal layer of epidermis, CD4+ T-cells and neighboring cells, and Pautrier’s microabscesses) were profiled for further assessment.

**Results and discussion:**

Interestingly, RUNX2, SHMT2, and MCM7 were upregulated in the enriched population of malignant T-cells in Pautrier’s microabscesses in patients who later developed advanced stages of disease. Expression of RUNX2, SHMT2 and MCM7 in malignant T-cells was confirmed in a subset of patients in MF skin using scRNA-seq datasets across multiple studies and correlating with stage of disease. Taken together, we provide first evidence that RUNX2 has potential as a biomarker to identify MF patients progressing to advanced stage disease. As RUNX2 has not previously been linked to MF, our data also shows the analytical strength of combining spatial transcriptomics with scRNA-seq analysis.

## 1 Introduction

Mycosis fungoides (MF) is the most common form of cutaneous T-cell lymphoma (CTCL), which is defined as non-Hodgkin lymphoma that presents in the skin without evidence of extracutaneous disease at the time of diagnosis (1). MF is histopathologically characterized by expansion of malignant skin homing CD4^+^ T-cells in the epidermal layer. However, a histopathologic diagnosis of MF can be difficult to obtain, as the histology of early MF can be non-specific and look like eczema or other inflammatory skin diseases. The clinical manifestations of MF can differ from patient to patient and rely to some extent on the malignant CD4^+^ T-cell clone and on the cellular and molecular composition of the surrounding microenvironment. Clinically, the diversity manifests in patients with respect to response to therapy and prognosis. MF ranges from localized early-stage disease (IA-IIA) to advanced stages (IIB-IV) with extracutaneous spread and distant metastases (2). In most patients, the disease evolves from few erythematous patches (patch stage) which can develop into more infiltrated plaques (plaque stage). In the patch and limited plaque stage, the prognosis is favorable. However, in a subset of patients, large and often ulcerated tumors (tumor stage) or erythroderma develop. Advanced stage MF is aggressive and associated with increased mortality (3).

MF associated mortality is highest during the first five years after diagnosis, indicating that a subset of patients has an aggressive form of MF (4). Five-year overall survival rates drop significantly with advanced stage disease from 85.8% and 62.2% in stage IB and IIB, respectively, to 23.3% in stage IVB (5). In recent years, improvements in diagnostic tools and treatment modalities have led to better outcomes for patients with MF, and it is evident that age at diagnosis, disease-propagation, treatment, and prolongation of time-to-progression plays a significant role in outcome for these patients (3). Despite prior emerging prognostic markers in MF (4, 6–12), it is still not possible to risk-stratify patients with newly-diagnosed MF in a clinical setting, nor to differentiate the subset of patients at increased risk of developing advanced stage disease. Conventional diagnostic tools, including immunohistochemistry (IHC), bulk DNA sequencing, or RNA expression analysis, do not measure intra-patients and intra-tumor heterogeneity to its full extent, either because of limited plex or lack of spatial resolution. Results from digital spatial profiling can potentially suggest a biologic difference and potentially pinpoint a valuable marker of more indolent vs. aggressive behavior in MF.

Thus, the aim of this study was to investigate the applicability of spatial transcriptome profiling to detect differences in gene expression in the malignant CD4^+^ T-cells at time-of-diagnosis and compare data from two distinct groups: skin samples from MF patients who progressed to advanced stage disease versus MF patients who remained in early-stage disease.

## 2 Materials and methods

### 2.1 Patient cohort

Fifteen patients with MF, who were followed and treated from initial diagnosis (between 1994-2012) and until May 2022 (**Table 1**) at the Department of Dermatology, Aarhus University Hospital, were included in this study. The study was approved by the local ethical committee (1–10–72–91–13) and the Data Protection Agency (Datatilsynet 1-16-02-478-15).

**Table 1:** Demographic and clinical characteristics of the patient cohort.

	Non-progression									Mean ± SD	Progression						Mean ± SD
Patient no.	1	2	3	4	5	6	7	8	9	-	10	11	12	13	14	15	-
**Age, *yr***	71	66	84	82	43	75	64	71	51	67.4 ± 13.5	70	74	80	58	14	69	60.8 ± 24.1
**Gender**	F	M	M	M	M	M	M	M	M	–	M	F	M	F	M	M	–
**Ethnicity**	Danish	Danish	Danish	Danish	Danish	Danish	Danish	Danish	Danish	–	Danish	Danish	Danish	Danish	Danish	Danish	–
**Primary lesion**	Plaque	Plaque	Patch	Plaque	Plaque	Plaque	Plaque	Plaque	Plaque	–	Plaque	Plaque	Plaque	Plaque	Plaque	Patch	–
**Lymph-adenopathy**	No	No	No	Yes†	No	No	No	No	No	–	No	No	No	No	No	No	–
**Extracutaneous spread**	No	No	No	No	No	No	No	No	No	–	No	No	No	No	No	No	–
**CD30^+^ expression in the initial lesion**	No	No	No	No	No	No	No	No	No	–	No	No	No	No	No	No	–
**Stage at time of diagnosis**	IB	IA	IB	IIA	IA	IA	IB	IB	IB	–	IB	IB	IB	IB	IA	IB	–
**Treatment at time of diagnosis**	None	None	Topical steroids	Topical steroids	Topical steroids	None	Topical steroids	Topical steroids	None	–	Topical steroids	Topical steroids	None	Topical steroids	Topical steroids	Topical steroids	–
**Stage at progression**	NA	NA	NA	NA	NA	NA	NA	NA	NA	–	IIB	III	IVA	III	IIB	IIB	–
**Time to progression, *years***	NA	NA	NA	NA	NA	NA	NA	NA	NA	–	12.3	3.6	0.4	1.1	4.1	1.2	3.8 ± 4.4
**Time without progression, years**	16.6	22.2	9.3	25.6	18.6	27.2	17.2	20.3	20.6	19.3 ± 5.2	NA	NA	NA	NA	NA	NA	NA
**Comorbidities**	CLL	Asthma	HT, DVT, atrial fibrillation	None	DM2, adiposity	IHD	HT, arthrosis	DM2, HT, angina pectoris, asthma	None	–	AS	Dilated cardiomyopathy	HT, thyrotoxicosis,	DM2, hypercholesterolemia	None	HT, IHD, IC, AS, bronchitis	–
**Status**	Dead	Dead	Dead	Dead	Alive	Dead	Alive	Dead	Alive	–	Dead	Dead	Dead	Dead	Alive	Dead	–

CLL, chronic lymphocytic leukemia; HT, hypertension; DVT, deep venous thrombosis; DM2, diabetes mellitus type 2; AS, atherosclerosis; IC, intermittent claudication; IHD, ischemic heart disease.

†Not due to MF.

By individual-level linkage to the medical records, we identified and acquired the specific skin biopsy that first established the diagnosis of early-stage MF. There had to be visible Pautrier’s microabscesses (a cluster enriched with malignant CD4^+^ T-cells in the epidermis) as assessed by the pathologist at the time of diagnosis. From the patient files, we extracted important clinical variables with complete and long-term follow-up such as age, gender, ethnicity, clinical stage at time of diagnosis and at progression, treatment at time of diagnosis, comorbidities, CD30 status and extracutaneous spread (**Table 1**). The early MF diagnosis (stage IA-IIA) was confirmed by clinical as well as immuno- and histopathological findings referenced in the patient files. The patients were staged according to the International Society for Cutaneous Lymphomas/European Organization of Research and Treatment of Cancer proposal (13). In total, seventeen formalin-fixed, paraffin-embedded (FFPE) skin biopsies were acquired. Two patients had to be excluded due to exhaustion of the biopsy core. Disease progression was defined as progression from the early stages (IA-IIA) to the advanced stages of MF (stage IIB-IVB), because progression to advanced stages of MF significantly affects the prognosis (14). The patients were thus subdivided into two groups according to whether their disease progressed to advanced stages (stage ≥ IIB) or remained in the early stages (stage < IIB) during follow-up. Some patients were treated with topical steroids and none of the patients received phototherapy at the time of diagnosis.

### 2.2 Sample tissue features and selecting visualization markers

Pathological archives were searched for the initial biopsy which confirmed the diagnosis of MF and a medical record following treatment and status of progression. Serial sections of five µm thickness derived from FFPE tissue blocks were sectioned. One section was stained with hematoxylin and eosin (H&E) as previously described (15). Furthermore, a serial unstained section was mounted on a positively charged histology slide (Superfrost Plus microscope slides cat: 12-550-15, Fisher Scientific, Waltham, MA, USA) for analyses using the GeoMx Digital Spatial Profiling (DSP) technology (NanoString Technologies Inc., Seattle, USA) in order to perform spatial transcriptomics. H&E-stained slides were digitized for image analysis using the whole slide digital pathology scanner NanoZoomer 2.0-HT (RRID: SCR_021658, Hamamatsu Photonics K.K, Hamamatsu City, Japan) with a 20×objective. Identification of epidermis, dermis, Pautrier’s microabscesses and basal layer was scored with 100% consensus agreement by three observers (M.D., T.E., and L.I.).

### 2.3 Preparation of samples for DSP

The process began with the deparaffinization of slides, followed by antigen retrieval. Subsequently, the samples underwent treatment with proteinase K (1 ug/ml for 15 minutes). Next, the slides were incubated overnight with DSP Barcoded RNA (indexing RNA oligos) prior to being stained with fluorescently conjugated antibodies to CD4, PanCK and DNA (**Supplementary Table S1**) for one hour. The slides were then washed in 2x saline-sodium citrate. Slides were introduced in the DSP instrument and submerged/washed in PBS with 0.1% Tween 20 as described in the NanoString Protocol.

### 2.4 ROI placement and compartmentalization/segmentation strategies

**Figure 1:**
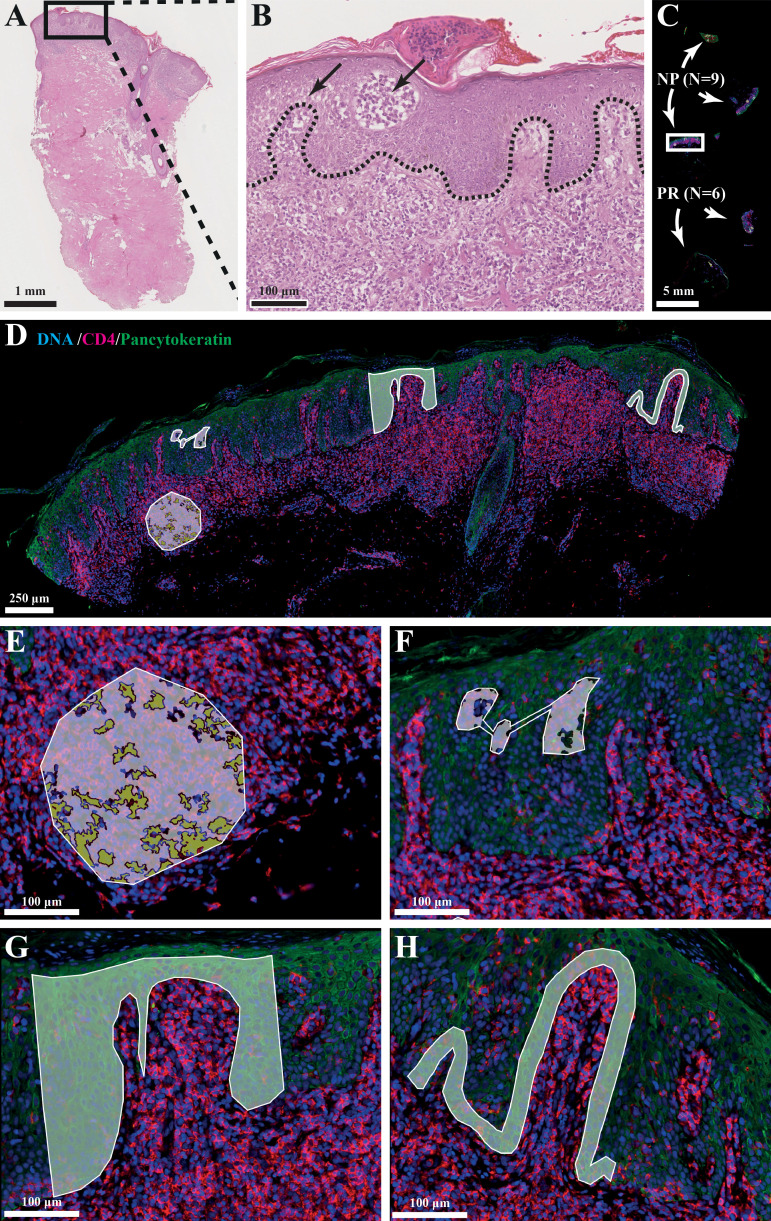
**(A, B)** Histological hallmarks of MF: intraepidermal vesicles (Pautrier’s microabscesses) and epidermotropism (atypical lymphocytes palisade along dermal-epidermal junction). **(C)** Slide setup with number of biopsies and type (NP=non-progression, PR=progression). **(D)** Photo of selected ROI/AOIs from one tissue sample. **(E)** ROI: dermal infiltrate, AOI: segmented into +/- CD4^+^ cells. **(F)** ROI: Pautrier’s microabscess. **(G)** ROI: Block of epidermis. **(H)** ROI: Basal layer.

Fluorescence scans (x20) were performed to obtain a high-resolution image of the tissue (**Figure 1**). Fluorescence scans were also imported into QuPath (RRID: SCR_018257, version 0.3.2) (16) which allowed for image analysis and cell counting. Regions-of-interest (ROIs) were selected in the DSP by matching with the previous selected regions on the H&E-stained serial section. Up to four ROIs were chosen per tissue consisting of the dermis, Pautrier’s microabscesses, the basal layer and a part of epidermis (**Figures 1E–H**). ROIs in the dermis were segmented into CD4^+^ T-cells and the surrounding tumor microenvironment.

### 2.5 Library preparation and sequencing

Indexing RNA oligos (probes), marked with a DSP barcode, were released from specific ROIs using UV light. Subsequently, 10 µl of liquid from above the ROI was gathered via a microcapillary tip and deposited into a 96-well plate. The probes underwent quantification through Next-Generation-Sequencing (NGS), wherein the resulting read numbers for each probe were then associated with the expression level of their corresponding gene targets.

To preserve ROI identity, indexing RNA oligos from each ROI were amplified via PCR using primers that hybridized to constant regions and contained distinct dual-indexed barcoding sequences. The PCR products underwent pooling and double purification using Ampure XP beads. The concentration and purity of the library were determined using a high-sensitivity DNA Bioanalyzer chip. Sequencing, involving paired-end reads (2x75 bp), was conducted on an Illumina MiSeq instrument.

### 2.6 Data processing

Count data files (dcc files) were produced from NGS fastq files using GeoMx HGS Pipeline software from NanoString. Subsequent data analysis including graphical presentations were made using the R statistical programming language and the packages NanoStringNCTools, GeomxTools and GeoWorkflows (17–19). Fraction of trimmed, stitched, and aligned reads were above 80% for all segments. Sequencing saturation was above 90% for all segments. A segment cutoff range based on biological diversity of 2% was applied, leaving 7,712 genes above the threshold.

### 2.7 Normalization

Third quartile (Q3) normalization was used prior to downstream visualization and differential expression analysis. Data were stratified according to cell type and five separate linear mixed models for the basal layer, cd4_dermis, cd4_dermis_micro, cd4_microabscess and epidermis were applied to contrast the progression variable with the scan id variable as a random factor. In addition, two models were made to combine evidence across different cell types. For both models, cell types were used as random effect factor.

Volcano plots were used for interpreting differential gene expression results, and the top five up- and downregulated genes for all models were reviewed in the literature. In addition, a non-exhaustive list of MF defining genes was added (**Supplementary Table S2**).

### 2.8 Relevance of spatial RNA profiling using single-cell RNA sequencing

Count matrices from six studies were obtained or generated from the Gene Expression Omnibus [accession numbers GSE182861 (20), GSE165623 (21), GSE173205 (22), and GSE128531 (23)], Sequence Read Archive (SRP332550 (24)), or Genome Sequence Archive (HRA000166 (25)). Upregulated genes from patients with progression in the Pautrier’s microabscess model were compared to findings in these scRNA-seq studies. Details on processing, integration, and analysis of scRNA-seq data can be found in **Supplementary Methods**.

## 3 Results

To establish the similarity between the histopathology of the non-progression group and the progression group we performed manual cell assessments in the investigated compartments in the samples used for DSP (**Supplementary Figure S1**). No significant difference between the two groups for any of the assessments was observed.

### 3.1 Upregulation of specific genes in CD4^+^ T-cells from Pautrier’s microabscesses in MF patients with progression

**Figure 2:**
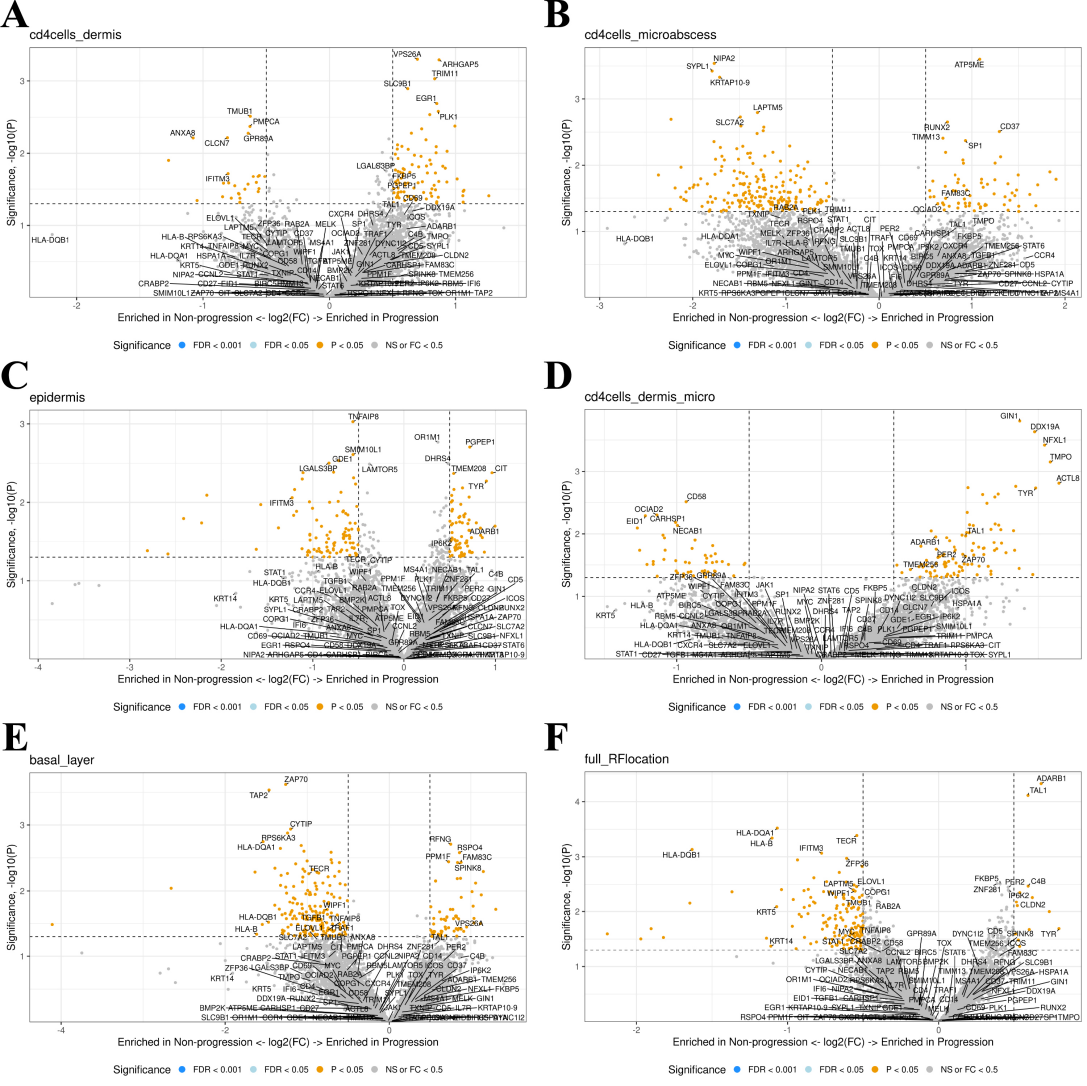
**(A–E)** A canonical visualization of the top five up- or downregulated genes for all five comparison models and known genes-of-interest (p<0.5) in progression vs. non-progression. None of the genes fell under the false detection rate (FDR) of 0.1. **(F)** All comparison models were combined in a total model showing the up- or downregulated genes enriched in progression vs. non-progression.

Next, we assessed the five different investigated compartments using DSP. The top five up- or downregulated genes for all five models and known genes-of-interest (**Supplementary Table S2**) in the progression vs. non-progression groups were labeled separately (**Figure 2**). None of the genes met the threshold of false detection rate (FDR) <0.1, nor were any genes differentially expressed when pooling all data and comparing progression vs. non-progression. All top five up- or downregulated genes were reviewed in the literature to identify candidate markers of progression. Comparison within the Pautrier’s microabscesses ensured expression data from clusters of cells enriched with malignant CD4^+^ T-cells (**Figure 2B**). Comparing this compartment in MF patients with progression vs. non-progression, we found upregulation of *RUNX2* (p=.002, FDR=.91), *ATP5ME* (p=.000, FDR=.99), *CD37* (p=.003, FDR=.99), *TIMM13* (p=.004, FDR=.99), *LPIN3* (p=.006, FDR=.99), *SHMT2* (p=.014, FDR=.99), *SP1* (p=.004, FDR=.99), *UPF2* (p=.008, FDR=.99), *AKAP1* (p=.009, FDR=.99), *SLC16A3* (p=.009, FDR=.99), *MCM7* (p=.013, FDR=.99), *PSTPIP1* (p=.014, FDR=.99), *PRPF31* (p=.016, FDR=.99) and *ZNF419* (p=.007, FDR=.99) in malignant CD4^+^ T-cells in Pautrier’s microabscesses in MF patients with progression compared with the non-progression group (**Figure 2B**).

**Figure 3:**
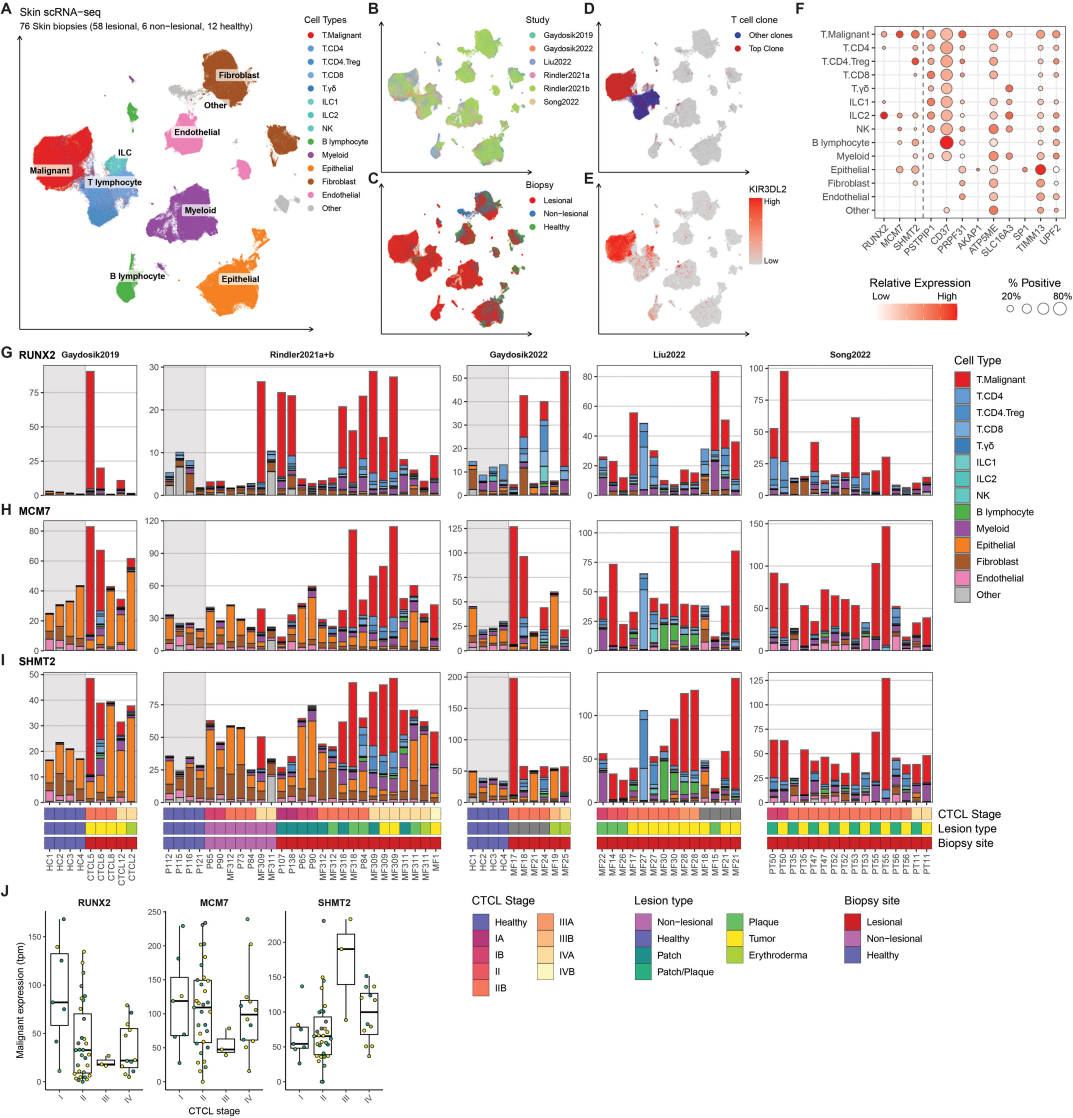
**(A–E)** Single-cell RNA sequencing (scRNA-seq) data from 76 skin biopsies (57 lesional, 7 non-lesional, 12 healthy) from 40 MF patients (stage I: six patients, stage II: 22 patients III: two patients, stage IV: eight patients, and two patients with unknown stage) and 12 healthy donors across six studies (20–25) were integrated using scANVI and visualized using Uniform Manifold Approximation and Projection (UMAP) colored by **(A)** cell types, **(B)** Study or origin, **(C)** Biopsy site, **(D)** Expression of top T cell receptor (TCR)-clone marking malignant cells and **(E)** expression of KIR3DL2 known to be high in malignant cells in CTCL. **(F)** scRNA-seq expression of genes upregulated in patients with progression in the Pautrier’s microabscess model shown as percent positive cells and mean expression across identified cell types. **(G–I)**. Transcript expression of the three selected genes (RUNX2, MCM7 and SHMT2) across each of the 76 Skin biopsies shown as transcripts per million (tpm) by sample. Bar height and colors indicate transcript contribution of each cell type population. **(J)** Mean malignant expression (as tpm) of RUNX2, MCM7 and SHMT2 divided into CTCL stage. Each dot represents mean malignant expression from one biopsy colored by lesion type. Bold horizontal line depicts median, box edges show 25^th^ and 75^th^ percentiles. The expression values indicated are gated on malignant T-cells only.

To validate expression of the progression-related genes within malignant T-cells from MF patients, we integrated and reanalyzed a collection of published scRNA-seq data sets containing a total of 64 skin samples (non-lesional/patch/plaque/tumor) from 40 patients with MF (stage I: six patients, stage II: 22 patients III: two patients, stage IV: eight patients, and two patients with unknown stage) and 12 healthy controls across six studies (20–25) (**Figure 3**). T-cell receptor clonality and the expression of malignant-associated genes were used to distinguish between malignant and nonmalignant T-cells (**Figures 3D, E**). For use as progression biomarkers, we focused on *RUNX2*, *MCM7* and *SHMT2* that all exhibited high expression within malignant cells and limited expression among other abundant cell types (**Figure 3F**). Interestingly, *RUNX2* was also highly expressed in type 2 innate lymphoid cells (**Figure 3F**). Supporting that *RUNX2* expression within Pautrier’s microabscesses in patients that progress was derived from malignant T-cells, we found that *RUNX2* was abundantly and almost exclusively found within malignant T-cells from MF patients and largely absent in other cell types and healthy controls (**Figure 3G**). *MCM7* and *SHMT2* transcripts were enriched within malignant T-cells, but also found among other cell types in both MF patients and healthy donors (**Figures 3H, I**). Supporting the potential utility as an early marker of MF patients that will progress, which only occurs in a subset of patients, we found heterogeneous expression of *RUNX2* in malignant T-cells between different patients at the early (I-II) stages as well as an inverse correlation with overall CTCL stage (**Figure 3J**). Similar patterns were less clear with *MCM7* and *SHMT2*.

Taken together, these data confirm that *RUNX2* is highly expressed by malignant CD4^+^ T-cells in a subset of MF patients at early CTCL stages, and higher *RUNX2* expression was detected in Pautrier’s microabscesses in early-stage disease in patients who progressed to advanced stages.

## 4 Discussion

The prognosis of patients with advanced MF is poor. This calls for a biologic marker which preferably at time of diagnosis, can identify patients progressing to advanced stage MF.

In the current study, gene expression data from spatial profiling of skin biopsies from patients who progressed to advanced stage disease were compared with patients who remained in early-stage disease. Current methods often rely on analyzing bulk samples which may dilute spatial compartments in the skin. However, spatial transcriptomic methods could help elucidate areas with known enriched pathological cells, discovering disease specific differences that so far have been hidden (26). Interestingly, our analyses provided multiple genes of interest. From the comparative model of Pautrier’s microabscesses, the gene expression of the transcription factor *RUNX2* was found to be upregulated differentially at time of diagnosis in patients who progressed to advanced stage disease. *RUNX2* is a member of the *RUNX* family of transcription factors and encodes a nuclear protein with a Runt DNA-binding domain. This protein is essential for osteoblastic differentiation and skeletal morphogenesis and acts as a scaffold for nucleic acids and regulatory factors involved in skeletal gene expression (27, 28). Interestingly, *RUNX2* is connected to the *RUNX3* gene which is already known to be dysregulated in cutaneous T-cell lymphoma (29), emphasizing the necessity of looking further into this gene as a potential biomarker. Prior experimental studies have found an association between *RUNX2* and other T-cell cancers (30–32). In other malignancies, *RUNX2* has been shown to drive expression of genes and pathways involved in invasion, angiogenesis, and metastasis such as *MMP9, VEGF, IL-8, TGFβ*, and *AKT/PI3K* (33), all of which have been reported as overexpressed by malignant T-cells from MF patients (34). Of notice, ectopic *RUNX2* expression has also been linked to genomic instability and drug resistance in cancer (35–37). Thus, it seems likely that an increased *RUNX2* expression by malignant T-cells at the time of diagnosis – as reported here – is not only an important novel biomarker but also a putative molecular driver of disease progression in MF. While *RUNX2* may be a driver of malignant transformation at the time of diagnosis it may act differently at more advanced stages as we observed a trend towards a lower concentration. Accordingly, the upregulation of *RUNX2* needs to be validated in a bigger cohort. One of the weaknesses in this study is the relatively high FDR values which thereby indicate a higher proportion of false positives among the genes identified as significantly differentially expressed. One possible explanation for the high FDR values in our samples could be due to poor RNA quality of our FFPE samples, which may be attributed to their age, with some samples being 20-25 years old. Lastly, from the assumption that a marker of progression will be greatly expressed in later stages of disease as compared to early-stage, *MCM7* involved in DNA replication, and *SHMT2* involved in metabolism are also interesting candidates for further investigation (38). Both genes have a lower expression in scRNA-seq in healthy skin as compared with malignant T-cells and *MCM7* expression exhibited a clear correlation with stage of disease. Moreover, increased levels of *MCM7* or *SHMT2* expression have been associated with poor prognosis in various cancers, including breast and lung cancers (39–41). It is notable that scRNA-seq analysis revealed high expression of *RUNX2* in type 2 innate lymphoid cells, indicating the need for further investigation of this cell type in MF.

MF can progress to high-grade large cell lymphoma which may be characterized by the increased expression of CD30 (42). A recent study utilized DSP to investigate CD30^+^ transformed MF and primary cutaneous anaplastic large-cell lymphoma (43). It was demonstrated that CD30^+^ tumor areas in MF with large-cell transformation had an increased concentration of macrophages and fibroblasts and a decreased concentration of regulatory T-cells compared with primary cutaneous anaplastic large-cell lymphoma. *MMP9*, a marker of cell-cell membrane interaction, was posited as a potential novel target in advanced MF (43). Classic Hodgkin lymphoma is also characterized by CD30^+^ malignant cells in the lymph nodes. In a recent study CD4^+^ T-cells close to and at 20 μm distance from CD30^+^ cells were profiled using DSP. *PD-1* and *CTLA-4* were significantly upregulated in cells close to CD30^+^ cells compared with cells at a distance from CD30^+^ cells (44). Both studies included relatively few patients, and none of the patients in our cohort were CD30^+^. However, both studies illustrate the good potential of DSP in biomarker identification in cutaneous lymphomas.

In conclusion, the present study identifies putative novel biomarkers for early detection of disease progression in patients with cutaneous lymphoma.

## Data Availability

The datasets used and analyzed during the current study are available at hosted at GEO repository (https://www.ncbi.nlm.nih.gov/geo/, accession number GSE275677) and the corresponding author on reasonable request.
