# Supplementary material for: RUNX2 as a novel biomarker for early identification of patients progressing to advanced-stage mycosis fungoides
Source: Front Oncol. 2024 Oct 7;14:1421443. doi: 10.3389/fonc.2024.1421443 (PMC11491341; doi:10.3389/fonc.2024.1421443)
Supplement: Supplementary file 1 [file DataSheet1.pdf]

Supplementary Material

1 Supplementary Data

1.1 Supplementary Methods

1.1.1 Single-cell RNA sequencing processing, integration, and analysis

Low quality cells were filtered when having less than 300 expressed genes or more than 50% mitochondrial transcripts, or if part of a cluster with uniformly low gene detection and high mitochondrial transcripts. Malignant cells were identified based on expression of a major expanded TCR clonotype (from scTCR-seq data) concurrent with high expression of malignant-associated genes (such as KIR3DL2 and TOX). Samples were initially batch-corrected and integrated using scVI[39] using expression of the top 3000 variable genes plus the 12 genes-of-interest from GeoMx analysis. Subsequent semi-supervised integration providing only Malignant cell label was done using scANVI[40]. Further malignant cells were then inferred based on co-clustering with TCR-defined malignant cells and expression of T cell- and malignant-associated genes in the absence of any scTCR-seq clonotype (i.e., due to drop out). Cell types were manually annotated based on expression of signature genes. For sample-wise comparisons, expression was quantified as transcripts per million (tpm) by dividing the UMI count for a given gene within a given population with the total UMI count from the sample after filtering and multiplied by 10^6.

1.2 Supplementary Tables

Marker	Cell	Clone	Lot	Dilution	Incubation length	Vendor	Isotype	Cat no.	RRID	Fluorophore
PanCK	Epithelial	AE1+AE3	211022-01	As provided by NanoString	60 min	Novus	Mouse Monoclonal, IgG1Kappa/IgG1 Kappa	NBP2-33200	AB_2924722	Alexa Fluor® 532
CD4	Helper T-cell	EPR6855	GR3424026	1:200	60 min	Abcam	Rabbit monoclonal, IgG	ab196147	AB_2923526	Alexa Fluor® 647
DNA	Nuclei	Syto13	-	As provided by NanoString	60 min	NanoString	-	As provided by NanoString	-	Alexa Flour® 525

Supplementary Table 1. List of morphology markers used in the experiment.

Number	MF biomarker	Expected expression in MF	Reference
1	BIRC5	High	Motamedi, Melika et al. “Patterns of Gene Expression in Cutaneous T-Cell Lymphoma: Systematic Review of Transcriptomic Studies in Mycosis Fungoides.” Cells vol. 10,6 1409. 6 Jun. 2021, doi:10.3390/cells10061409
2	BLK	High	Petersen DL, Krejsgaard T, Berthelsen J, et al. B-lymphoid tyrosine kinase (Blk) is an oncogene and a potential target for therapy with dasatinib in cutaneous T-cell lymphoma (CTCL). Leukemia. 2014;28(10):2109-2112. doi:10.1038/leu.2014.192
3	BMP2K	High vs normal skin	Humme D, Haider A, Möbs M, et al. Aurora Kinase A Is Upregulated in Cutaneous T-Cell Lymphoma and Represents a Potential Therapeutic Target. J Invest Dermatol. 2015;135(9):2292-2300. doi:10.1038/jid.2015.139
4	BTSE1	High	-
5	CCNL2	Low vs normal skin	Motamedi M, Xiao MZX, Iyer A, Gniadecki R. Patterns of Gene Expression in Cutaneous T-Cell Lymphoma: Systematic Review of Transcriptomic Studies in Mycosis Fungoides. Cells. 2021;10(6):1409. Published 2021 Jun 6. doi:10.3390/cells10061409
6	CCR3	High expression is associated with poor prognosis	Shono Y, Suga H, Kamijo H, et al. Expression of CCR3 and CCR4 Suggests a Poor Prognosis in Mycosis Fungoides and Sézary Syndrome. Acta Derm Venereol. 2019;99(9):809-812. doi:10.2340/00015555-3207
7	CCR4	High expression is associated with poor prognosis	Shono Y, Suga H, Kamijo H, et al. Expression of CCR3 and CCR4 Suggests a Poor Prognosis in Mycosis Fungoides and Sézary Syndrome. Acta Derm Venereol. 2019;99(9):809-812. doi:10.2340/00015555-3207
8	CD3	High/Abnormal, predictor of severe disease and poor prognosis	Vermeer MH, Moins-Teisserenc H, Bagot M, Quaglino P, Whittaker S. Flow cytometry for the assessment of blood tumour burden in cutaneous T-cell lymphoma: towards a standardized approach. Br J Dermatol. 2022;187(1):21-28. doi:10.1111/bjd.21053
9	CD4	High	Vermeer MH, Moins-Teisserenc H, Bagot M, Quaglino P, Whittaker S. Flow cytometry for the assessment of blood tumour burden in cutaneous T-cell lymphoma: towards a standardized approach. Br J Dermatol. 2022;187(1):21-28. doi:10.1111/bjd.21053
10	CD5	Low	Ryu HJ, Kim SI, Jang HO, et al. Evaluation of the International Society for Cutaneous Lymphoma Algorithm for the Diagnosis of Early Mycosis Fungoides. Cells. 2021;10(10):2758. Published 2021 Oct 15. doi:10.3390/cells10102758

11	CD7	Low	Vermeer MH, Moins-Teisserenc H, Bagot M, Quaglino P, Whittaker S. Flow cytometry for the assessment of blood tumour burden in cutaneous T-cell lymphoma: towards a standardized approach. Br J Dermatol. 2022;187(1):21-28. doi:10.1111/bjd.21053
12	CD8	Lower compared with CD4	-
13	CD14	Upregulated protein in early-stage MF	Leng L, Liu Z, Ma J, et al. Proteomic identification of new diagnostic biomarkers of early-stage cutaneous mycosis fungoides. Cancer Commun (Lond). 2022;42(6):558-562. doi:10.1002/cac2.12266
14	CD26	Low	Vermeer MH, Moins-Teisserenc H, Bagot M, Quaglino P, Whittaker S. Flow cytometry for the assessment of blood tumour burden in cutaneous T-cell lymphoma: towards a standardized approach. Br J Dermatol. 2022;187(1):21-28. doi:10.1111/bjd.21053
15	CD27	Higher in MF compared with healthy skin	Galliano I, Daprà V, Ponti R, et al. CD27 mRNA expression in mycosis fungoides. Ital J Dermatol Venerol. 2022;157(3):275-280. doi:10.23736/S2784-8671.21.06953-X
16	CD30	High expression associated with progression	Edinger JT, Clark BZ, Pucevich BE, Geskin LJ, Swerdlow SH. CD30 expression and proliferative fraction in nontransformed mycosis fungoides. Am J Surg Pathol. 2009;33(12):1860-1868. doi:10.1097/PAS.0b013e3181bf677d
17	CD45RA	Highly expressed	Mehdi SJ, Moerman-Herzog AM, Wong HK. Isolating Human Peripheral Blood Mononuclear Cells and CD4+ T cells from Sézary Syndrome Patients for Transcriptomic Profiling. J Vis Exp. 2021;(176):10.3791/61470. Published 2021 Oct 14. doi:10.3791/61470
18	CD45RO	Highly expressed	Mehdi SJ, Moerman-Herzog AM, Wong HK. Isolating Human Peripheral Blood Mononuclear Cells and CD4+ T cells from Sézary Syndrome Patients for Transcriptomic Profiling. J Vis Exp. 2021;(176):10.3791/61470. Published 2021 Oct 14. doi:10.3791/61470
19	CD68	Higher in aggressive MF	Atzmony L, Moyal L, Feinmesser M, et al. Stage-dependent Increase in Expression of miR-155 and Ki-67 and Number of Tumour-associated Inflammatory Cells in Folliculotropic Mycosis Fungoides. Acta Derm Venereol. 2020;100(15):adv00230. Published 2020 Aug 17. doi:10.2340/00015555-3578
20	CD69	Lower as lesion progresses	Rindler K, Jonak C, Alkon N, et al. Single-cell RNA sequencing reveals markers of disease progression in primary cutaneous T-cell lymphoma. Mol Cancer. 2021;20(1):124. Published 2021 Sep 28. doi:10.1186/s12943-021-01419-2

21	CLA	Associated with CD4+ T-cells	Sokolowska-Wojdylo M, Wenzel J, Gaffal E, et al. Circulating clonal CLA(+) and CD4(+) T cells in Sezary syndrome express the skin-homing chemokine receptors CCR4 and CCR10 as well as the lymph node-homing chemokine receptor CCR7. Br J Dermatol. 2005;152(2):258-264. doi:10.1111/j.1365-2133.2004.06325.x
22	COL18A1	Upregulated protein in early-stage MF	Leng L, Liu Z, Ma J, et al. Proteomic identification of new diagnostic biomarkers of early-stage cutaneous mycosis fungoides. Cancer Commun (Lond). 2022;42(6):558-562. doi:10.1002/cac2.12266
23	CRABP2	Downregulated	Leng L, Liu Z, Ma J, et al. Proteomic identification of new diagnostic biomarkers of early-stage cutaneous mycosis fungoides. Cancer Commun (Lond). 2022;42(6):558-562. doi:10.1002/cac2.12266
24	CXCR4	Lower as lesion progresses	Rindler K, Jonak C, Alkon N, et al. Single-cell RNA sequencing reveals markers of disease progression in primary cutaneous T-cell lymphoma. Mol Cancer. 2021;20(1):124. Published 2021 Sep 28. doi:10.1186/s12943-021-01419-2
25	DEPDC	Upregulated	Motamedi, Melika et al. “Patterns of Gene Expression in Cutaneous T-Cell Lymphoma: Systematic Review of Transcriptomic Studies in Mycosis Fungoides.” Cells vol. 10,6 1409. 6 Jun. 2021, doi:10.3390/cells10061409
26	DYNC1I2	Upregulated	Leng L, Liu Z, Ma J, et al. Proteomic identification of new diagnostic biomarkers of early-stage cutaneous mycosis fungoides. Cancer Commun (Lond). 2022;42(6):558-562. doi:10.1002/cac2.12266
27	HN1	Upregulated	Motamedi, Melika et al. “Patterns of Gene Expression in Cutaneous T-Cell Lymphoma: Systematic Review of Transcriptomic Studies in Mycosis Fungoides.” Cells vol. 10,6 1409. 6 Jun. 2021, doi:10.3390/cells10061409
28	HSPA1A	Downregulated	Rindler K, Jonak C, Alkon N, et al. Single-cell RNA sequencing reveals markers of disease progression in primary cutaneous T-cell lymphoma. Mol Cancer. 2021;20(1):124. Published 2021 Sep 28. doi:10.1186/s12943-021-01419-2
29	ICOS	High	Di Raimondo C, Rubio-Gonzalez B, Palmer J, et al. Expression of immune checkpoint molecules programmed death protein 1, programmed death-ligand 1 and inducible T-cell co-stimulator in mycosis fungoides and Sézary syndrome: association with disease stage and clinical outcome. Br J Dermatol. 2022;187(2):234-243. doi:10.1111/bjd.21063
30	IFI6	Upregulated	Motamedi, Melika et al. “Patterns of Gene Expression in Cutaneous T-Cell Lymphoma: Systematic Review of

			Transcriptomic Studies in Mycosis Fungoides.” Cells vol. 10,6 1409. 6 Jun. 2021, doi:10.3390/cells10061409
31	IL-4	Upregulated in progressed stages	Asadullah K, Döcke WD, Haeussler A, Sterry W, Volk HD. Progression of mycosis fungoides is associated with increasing cutaneous expression of interleukin-10 mRNA. J Invest Dermatol. 1996;107(6):833-837. doi:10.1111/1523-1747.ep12330869
32	IL-5	Upregulated	Nielsen M, Nissen MH, Gerwien J, et al. Spontaneous interleukin-5 production in cutaneous T-cell lymphoma lines is mediated by constitutively activated Stat3. Blood. 2002;99(3):973-977. doi:10.1182/blood.v99.3.973
33	IL7R	Downregulated	Rindler K, Jonak C, Alkon N, et al. Single-cell RNA sequencing reveals markers of disease progression in primary cutaneous T-cell lymphoma. Mol Cancer. 2021;20(1):124. Published 2021 Sep 28. doi:10.1186/s12943-021-01419-2
34	IL-13	Upregulated	Geskin, Larisa J et al. “Interleukin-13 is overexpressed in cutaneous T-cell lymphoma cells and regulates their proliferation.” Blood vol. 125,18 (2015): 2798-805. doi:10.1182/blood-2014-07-590398
35	IL15	Upregulated	Asadullah K, Haeussler-Quade A, Gellrich S, et al. IL-15 and IL-16 overexpression in cutaneous T-cell lymphomas: stage-dependent increase in mycosis fungoides progression. Exp Dermatol. 2000;9(4):248-251. doi:10.1034/j.1600-0625.2000.009004248.x
36	JAK1	Deregulated	Pérez, Cristina et al. “Mutated JAK kinases and deregulated STAT activity are potential therapeutic targets in cutaneous T-cell lymphoma.” Haematologica vol. 100,11 (2015): e450-3. doi:10.3324/haematol.2015.132837
37	JAK2	Deregulated	Deregulated
38	Ki67	Positive correlation with the clinical stage of MF	Gambichler T, Bischoff S, Bechara FG, Altmeyer P, Kreuter A. Expression of proliferation markers and cell cycle regulators in T cell lymphoproliferative skin disorders. J Dermatol Sci. 2008;49(2):125-132. doi:10.1016/j.jdermsci.2007.07.011
39	KIR3DL2	Upregulated	Sun J, Wang Y. KIR3DL2 in cutaneous T-cell lymphoma: from a promising biomarker to a potential therapeutic target. Br J Dermatol. 2020;182(6):1325-1326. doi:10.1111/bjd.18682
40	KRT5	Upregulated in early-stage MF	Leng L, Liu Z, Ma J, et al. Proteomic identification of new diagnostic biomarkers of early-stage cutaneous mycosis fungoides. Cancer Commun (Lond). 2022;42(6):558-562. doi:10.1002/cac2.12266
41	KRT14	Upregulated in early-stage MF	Leng L, Liu Z, Ma J, et al. Proteomic identification of new diagnostic biomarkers of early-stage cutaneous

			mycosis fungoides. Cancer Commun (Lond). 2022;42(6):558-562. doi:10.1002/cac2.12266
42	MELK	Upregulated	Motamedi M, Xiao MZX, Iyer A, Gniadecki R. Patterns of Gene Expression in Cutaneous T-Cell Lymphoma: Systematic Review of Transcriptomic Studies in Mycosis Fungoides. Cells. 2021;10(6):1409. Published 2021 Jun 6. doi:10.3390/cells10061409
43	MS4A1	Upregulated in MF compared with controls Upregulated in disease progression	Nielsen PR, Eriksen JO, Sørensen MD, et al. Role of B-cells in Mycosis Fungoides. Acta Derm Venereol. 2021;101(3):adv00413. Published 2021 Mar 11. doi:10.2340/00015555-3775
44	MYC	Upregulated	Xiao MZX, Hennessey D, Iyer A, et al. Transcriptomic changes during stage progression of mycosis fungoides. Br J Dermatol. 2022;186(3):520-531. doi:10.1111/bjd.20760
45	NPHP3	Downregulated	Motamedi M, Xiao MZX, Iyer A, Gniadecki R. Patterns of Gene Expression in Cutaneous T-Cell Lymphoma: Systematic Review of Transcriptomic Studies in Mycosis Fungoides. Cells. 2021;10(6):1409. Published 2021 Jun 6. doi:10.3390/cells10061409
46	OX40	Positively correlated with disease severity	Kawana Y, Suga H, Kamijo H, Miyagaki T, Sugaya M, Sato S. Roles of OX40 and OX40 Ligand in Mycosis Fungoides and Sézary Syndrome. Int J Mol Sci. 2021;22(22):12576. Published 2021 Nov 22. doi:10.3390/ijms222212576
47	OX40L	Positively correlated with disease severity	Kawana Y, Suga H, Kamijo H, Miyagaki T, Sugaya M, Sato S. Roles of OX40 and OX40 Ligand in Mycosis Fungoides and Sézary Syndrome. Int J Mol Sci. 2021;22(22):12576. Published 2021 Nov 22. doi:10.3390/ijms222212576
48	P63	Upregulated	Chavan RN, Bridges AG, Knudson RA, et al. Somatic rearrangement of the TP63 gene preceding development of mycosis fungoides with aggressive clinical course. Blood Cancer J. 2014;4(10):e253. Published 2014 Oct 17. doi:10.1038/bcj.2014.73
49	PD-1	Upregulated	Cetinözman F, Jansen PM, Vermeer MH, Willemze R. Differential expression of programmed death-1 (PD-1) in Sézary syndrome and mycosis fungoides. Arch Dermatol. 2012;148(12):1379-1385. doi:10.1001/archdermatol.2012.2089
50	PD-L1	Upregulated	Beygi S, Fernandez-Pol S, Duran G, et al. Pembrolizumab in mycosis fungoides with PD-L1 structural variants. Blood Adv. 2021;5(3):771-774. doi:10.1182/bloodadvances.2020002371

51	PLK1	Upregulated	Motamedi M, Xiao MZX, Iyer A, Gniadecki R. Patterns of Gene Expression in Cutaneous T-Cell Lymphoma: Systematic Review of Transcriptomic Studies in Mycosis Fungoides. Cells. 2021;10(6):1409. Published 2021 Jun 6. doi:10.3390/cells10061409
52	RBM5	Downregulated	Motamedi M, Xiao MZX, Iyer A, Gniadecki R. Patterns of Gene Expression in Cutaneous T-Cell Lymphoma: Systematic Review of Transcriptomic Studies in Mycosis Fungoides. Cells. 2021;10(6):1409. Published 2021 Jun 6. doi:10.3390/cells10061409
53	SCCA1	Upregulated Positively correlated with disease severity	Oka K, Miyagawa T, Suga H, et al. Increased expression of squamous cell carcinoma antigen 1 and 2 in mycosis fungoides and Sézary syndrome. Increased expression of squamous cell carcinoma antigen 1 and 2 in mycosis fungoides and Sézary syndrome. Eur J Dermatol. 2022;32(4):464-470. doi:10.1684/ejd.2022.4305
54	SCCA2	Upregulated Positively correlated with disease severity	Oka K, Miyagawa T, Suga H, et al. Increased expression of squamous cell carcinoma antigen 1 and 2 in mycosis fungoides and Sézary syndrome. Increased expression of squamous cell carcinoma antigen 1 and 2 in mycosis fungoides and Sézary syndrome. Eur J Dermatol. 2022;32(4):464-470. doi:10.1684/ejd.2022.4305
55	SPRR1Av1	Upregulated Positively correlated with disease severity	Trzeciak M, Olszewska B, Sakowicz-Burkiewicz M, et al. Expression Profiles of Genes Encoding Cornified Envelope Proteins in Atopic Dermatitis and Cutaneous T-Cell Lymphomas. Nutrients. 2020;12(3):862. Published 2020 Mar 24. doi:10.3390/nu12030862
56	STAT1	Deregulated	Netchiporouk E, Litvinov IV, Moreau L, Gilbert M, Sasseville D, Duvic M. Deregulation in STAT signaling is important for cutaneous T-cell lymphoma (CTCL) pathogenesis and cancer progression. Cell Cycle. 2014;13(21):3331-3335. doi:10.4161/15384101.2014.965061
57	STAT6	Upregulated	Gaydosik AM, Queen DS, Trager MH, Akilov OE, Geskin LJ, Fuschiotti P. Genome-wide transcriptome analysis of the STAT6-regulated genes in advanced-stage cutaneous T-cell lymphoma. Blood. 2020;136(15):1748-1759. doi:10.1182/blood.2019004725
58	STING	Upregulated	Takayanagi-Hara R, Sawada Y, Sugino H, et al. STING expression is an independent prognostic factor in patients with mycosis fungoides. Sci Rep. 2022;12(1):12739. Published 2022 Jul 26. doi:10.1038/s41598-022-17122-1
59	TGFB1	Upregulated	Motamedi M, Xiao MZX, Iyer A, Gniadecki R. Patterns of Gene Expression in Cutaneous T-Cell Lymphoma: Systematic Review of Transcriptomic Studies in Mycosis Fungoides. Cells. 2021;10(6):1409. Published 2021 Jun 6. doi:10.3390/cells10061409

60	TNFSF11	Upregulated	Motamedi M, Xiao MZX, Iyer A, Gniadecki R. Patterns of Gene Expression in Cutaneous T-Cell Lymphoma: Systematic Review of Transcriptomic Studies in Mycosis Fungoides. Cells. 2021;10(6):1409. Published 2021 Jun 6. doi:10.3390/cells10061409
61	TOX	Upregulated	Pileri A, Cavicchi M, Bertuzzi C, et al. TOX Expression in Mycosis Fungoides and Sezary Syndrome. Diagnostics (Basel). 2022;12(7):1582. Published 2022 Jun 29. doi:10.3390/diagnostics12071582
62	TRAF1	Upregulated	Nielsen PR, Eriksen JO, Lindahl LM, et al. Diagnostic Two-Gene Classifier in Early-Stage Mycosis Fungoides: A Retrospective Multicenter Study. J Invest Dermatol. 2021;141(1):213-217.e5. doi:10.1016/j.jid.2020.04.026
63	TSC1	Downregulated	Motamedi M, Xiao MZX, Iyer A, Gniadecki R. Patterns of Gene Expression in Cutaneous T-Cell Lymphoma: Systematic Review of Transcriptomic Studies in Mycosis Fungoides. Cells. 2021;10(6):1409. Published 2021 Jun 6. doi:10.3390/cells10061409
64	TXNIP	Downregulated	Rindler K, Jonak C, Alkon N, et al. Single-cell RNA sequencing reveals markers of disease progression in primary cutaneous T-cell lymphoma. Mol Cancer. 2021;20(1):124. Published 2021 Sep 28. doi:10.1186/s12943-021-01419-2
65	ZFP36	Downregulated	Rindler K, Jonak C, Alkon N, et al. Single-cell RNA sequencing reveals markers of disease progression in primary cutaneous T-cell lymphoma. Mol Cancer. 2021;20(1):124. Published 2021 Sep 28. doi:10.1186/s12943-021-01419-2

Supplementary Table 2. Selection of mycosis fungoides defining genes.

1.3 Supplementary Figures

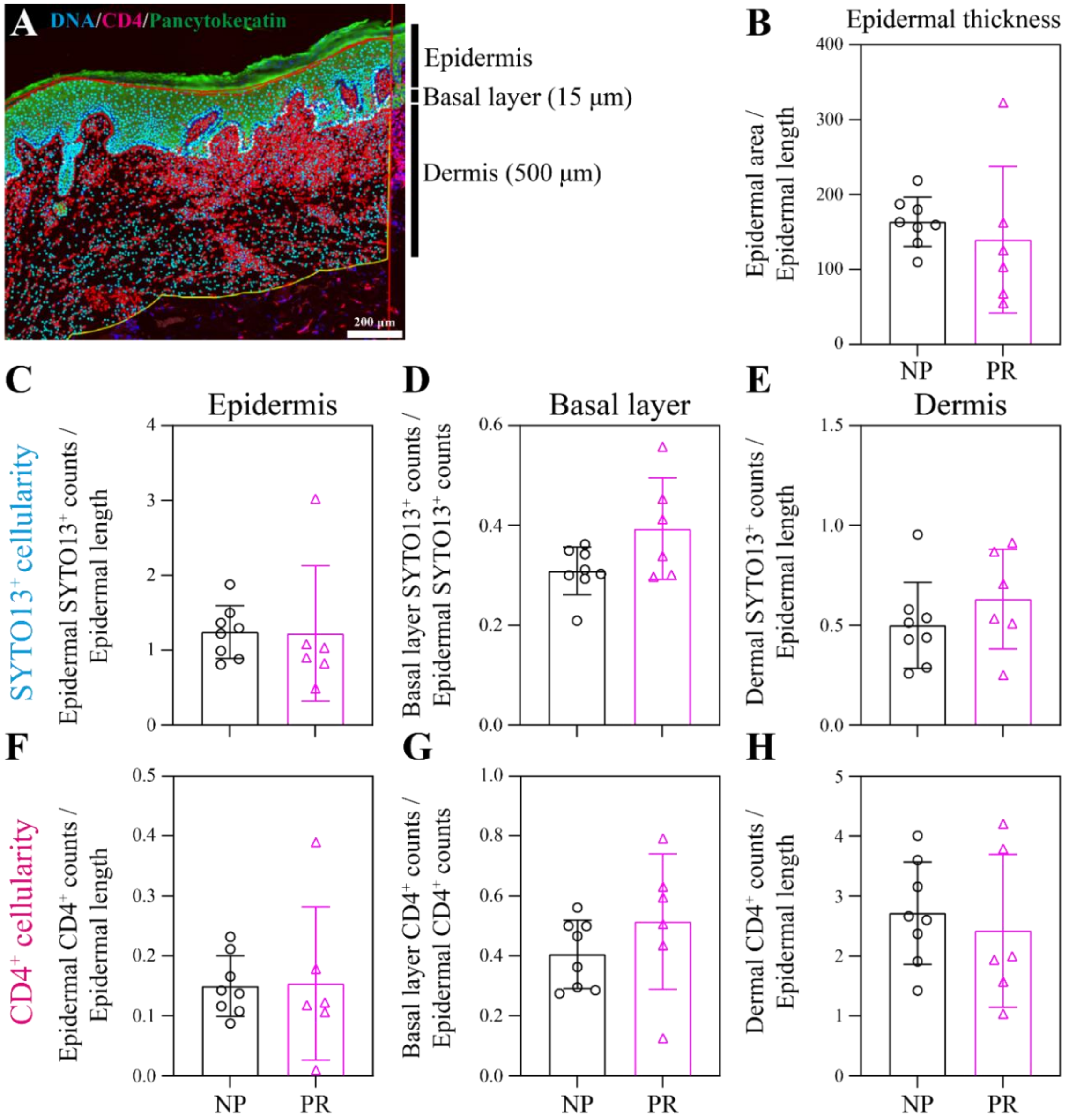

Supplementary Figure 1. Histopathology and cell counting analysis. A. Depiction of segments in which cell counts were performed delineating the epidermis, basal layer, and dermis. B. Epidermal thickness between non-progression (NP) and progression (PR) patients. C-E. SYTO13$^+$ cell counts (cellularity) divided by epidermal length (C), as a fraction of SYTO13+ cells in the basal layer out of all SYTO13$^+$ cells in the epidermis (D), and SYTO13$^+$ cells in the dermis divided by epidermal length (E). F-H. CD4$^+$ cell counts divided by epidermal length (F), as a fraction of CD4$^+$ cells in the basal layer out of all CD4$^+$ cells in the epidermis (G), and dermal cells divided by epidermal length (H). †Students t-test was used to assess significant differences however no comparison fulfilled a p-value <0.05.
